# Blast furnace slag-Mg(OH)_2_ cements activated by sodium carbonate

**DOI:** 10.1039/c8ra03717e

**Published:** 2018-06-26

**Authors:** Sam A. Walling, Susan A. Bernal, Laura J. Gardner, Hajime Kinoshita, John L. Provis

**Affiliations:** Immobilisation Science Laboratory, Department of Materials Science & Engineering, Sir Robert Hadfield Building, The University of Sheffield S1 3JD UK j.provis@sheffield.ac.uk +44 (0) 114 222 5490

## Abstract

The structural evolution of a sodium carbonate activated slag cement blended with varying quantities of Mg(OH)_2_ was assessed. The main reaction products of these blended cements were a calcium-sodium aluminosilicate hydrate type gel, an Mg-Al layered double hydroxide with a hydrotalcite type structure, calcite, and a hydrous calcium aluminate phase (tentatively identified as a carbonate-containing AFm structure), in proportions which varied with Na_2_O/slag ratios. Particles of Mg(OH)_2_ do not chemically react within these cements. Instead, Mg(OH)_2_ acts as a filler accelerating the hardening of sodium carbonate activated slags. Although increased Mg(OH)_2_ replacement reduced the compressive strength of these cements, pastes with 50 wt% Mg(OH)_2_ still reached strengths of ∼21 MPa. The chemical and mechanical characteristics of sodium carbonate activated slag/Mg(OH)_2_ cements makes them a potentially suitable matrix for encapsulation of high loadings of Mg(OH)_2_-bearing wastes such as Magnox sludge.

## Abstract 2

Novel cements can contain up to 50 wt% Mg(OH)_2_, offering a new route to immobilisation of this nuclear waste constituent.

## Graphical abstract

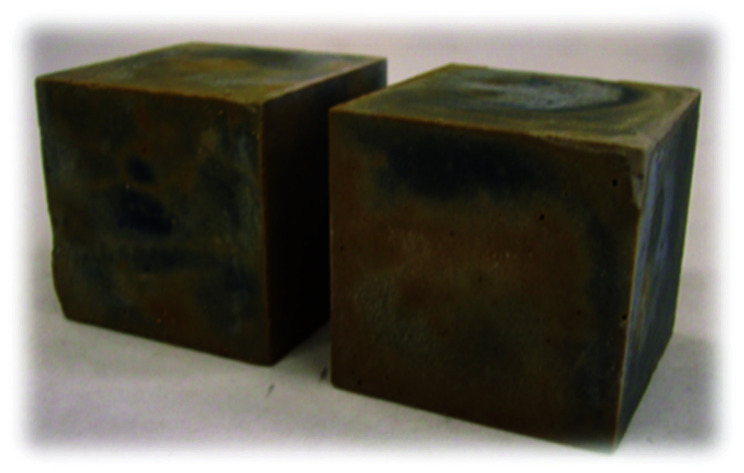

## 1. Introduction

Alkali-activated slag cements are Portland cement-free binders produced through the chemical reaction between an alkaline activator and ground granulated blast furnace slag (GGBS). The performance of these materials is strongly dependent on the mineralogy and composition of the slag, and the nature and concentration of the alkaline activator chosen,^1^ among other factors. Sodium carbonate-activated slags have attracted less attention than other alkali-activated slag cements which use sodium hydroxide or sodium silicate as the main activators, despite proven successful usage in Eastern Europe and the CIS as alternative cements for production of mortars and concretes.^2^ This is mainly because of the longer setting times identified in these binders (often >5 days),^3,4^ compared with cements produced using other alkali-activators.

The use of sodium carbonate as the activator for GGBS has advantages compared with sodium hydroxide or silicate solutions,^5^ as it is easier and safer to handle due to its powdered and less hygroscopic nature, and when dissolved in water will reach a lower pH than that of sodium hydroxide or silicate solutions with an equivalent content of alkalis.^3^ Recent studies have demonstrated^6,7^ that the often-observed delayed hardening of these cements is strongly dependent on the chemistry of the slag used, and therefore, the general assumption that setting time problems will occur when using this activator is misleading.

The UK has accumulated significant quantities of radioactive Mg(OH)_2_ sludges from its Magnox nuclear programme, which are classed as intermediate level waste (ILW) streams.^8^ These wastes consist of up to 100% sludge, predominantly Mg(OH)_2_ with some uranium metal or oxides, along with varying radionuclides from nuclear fission.^9,10^ Other ILW streams are processed by encapsulation/immobilisation in composite cements based on ordinary Portland cement, GGBS and pulverised fuel ash.^11^ This Mg(OH)_2_-rich sludge is more challenging to treat using such binders, due to the high water content of the waste.^12^ Equally, any alternative cementitious matrices which could make use of the abundant Mg within these wastes as part of the encapsulation process could potentially improve upon conventional wasteform properties.^13^

GGBS rich-binders are beneficial in the encapsulation of radioactive wastes, as the slag contains sulphide and potentially Fe(ii) which act as reducing agents, controlling the solubility of key radionuclides.^14,15^ The hydration of GGBS in cements also often forms layered double hydroxides with a hydrotalcite-type structure (general formula M_*x*_^2+^M_*y*_^3+^(OH)_2*x*+3*y*−*nz*_(A^*n*−^)_*z*_·*m*H_2_O, where A^*n*−^ is often OH^−^, Cl^−^ or CO_3_^2−^, and *x*/*y* is generally between 2 and 3 (ref. 16–18)), which is of particular interest for immobilisation of nuclear wastes due to its ability to retain certain long-lived radionuclides such as ^129^I and ^36^Cl (ref. 19) that are present in Magnox sludges.^20^

A high MgO content within GGBS favours hydrotalcite formation in sodium silicate-activated^21,22^ and sodium carbonate activated slags.^6^ The addition of either reactive MgO^23^ or calcined Mg-Al layered double hydroxides^6^ to alkali-activated slag binders can also favour the formation of larger amounts of hydrotalcite-group phases. Activated slag cements with increased contents of such phases appear to be more resistant to degradation by carbonation^22^ and chloride penetration,^24^ potentially opening the door to the development of more durable cementitious materials.

The effect of the addition of Mg(OH)_2_ to alkali-activated binders has not been reported in the open literature, although Collier *et al.*^25^ identified that the addition of Mg(OH)_2_ to Portland cement/slag cement composites potentially promoted the formation of higher contents of hydrotalcite. If this were to additionally hold true for Mg(OH)_2_ addition to alternative binders, then this could interact with the cement, enhancing hydration and forming part of the binding phase, increasing the achievable waste loadings in an immobilisation context.

In this study a sodium carbonate activated slag binder was blended with varying quantities of Mg(OH)_2_ (10, 30 and 50 wt% of the total binder). Kinetics of reaction were assessed *via* isothermal calorimetry, and hardened samples were evaluated using X-ray diffraction, Fourier transform infrared spectroscopy, thermogravimetry, solid state nuclear magnetic resonance, and scanning electron microscopy, focussing on both early age and longer-term aged samples. Particular emphasis is laid on exploring and understanding the binding gels within this system, and the degree to which Mg(OH)_2_ might have influenced their chemistries, to assess the level of structural substitution of Mg-containing phases that is possible.

## 2. Experimental programme

### 2.1. Materials

A ground granulated blast furnace slag (GGBS) supplied by Civil and Marine Ltd. was used in this study, the chemical composition of which is reported in Table 1. The GGBS had a particle size range between 0.5 and 59 μm, with *D*_50_ = 13.2 μm measured by laser particle analysis (Malvern Mastersizer 3000 in 2-propanol), and a measured Blaine fineness of 515.1 ± 1.1 m^2^ kg^−1^. The chemical composition in Table 1 gives a slag basicity coefficient (*K*_b_ = (CaO + MgO)/(SiO_2_ + Al_2_O_3_)^26,27^) of 1.01, indicating that this is a basic slag, with a quality coefficient (*Q*_c_ = (CaO + MgO + Al_2_O_3_)/(SiO_2_ + TiO_2_)^27^) of 1.70. Commercial magnesium hydroxide (Mg(OH)_2_, Alfa Aesar, >95% purity, Blaine fineness 1179.2 ± 5.9 m^2^ kg^−1^) was blended into the binder mixes, and as an alkaline activator reagent-grade sodium carbonate (Na_2_CO_3_ (>99.5% purity), Sigma-Aldrich) was used.

**Table 1:** Chemical composition of the GGBS determined by X-ray fluorescence (XRF)

Oxide	Quantity (wt%)
MgO	8.4
Al_2_O_3_	12.6
SiO_2_	35.4
CaO	40.3
TiO_2_	0.6
Others	2.48

### 2.2. Sample preparation

All binders were formulated with 10 g of Na_2_CO_3_ per 90 g of GGBS + Mg(OH)_2_ binder, with the proportion of Mg(OH)_2_:GGBS varied (Table 2). Although the overall Na_2_CO_3_ content remained constant, the equivalent ratio of Na_2_O to slag increased as more of the GGBS was replaced by Mg(OH)_2_.

**Table 2:** Formulation of sodium carbonate activated slag cements – weight percentage basis

Sample ID	Mg(OH)_2_	GGBS	Na_2_CO_3_	Water/solids ratio	g Na_2_O/100 g GGBS
M0	—	90	10	0.30	6.50
M10	10	80	10	0.35	7.31
M30	30	60	10	0.50	9.75
M50	50	40	10	0.60	14.62

Samples were prepared in a Kenwood benchtop mixer by first combining the required quantity of water and solid sodium carbonate. After dissolution of the solid Na_2_CO_3_, the GGBS was added to the mix over 2 minutes, followed by the addition of the Mg(OH)_2_ over a further 2 minutes of mixing. The amount of water added was increased commensurate with Mg(OH)_2_ addition, to maintain roughly consistent fluidity between samples (judged during mixing). The pastes for hardened-state analysis were cast in 50 mL centrifuge tubes and stored at 20 °C and >90% relative humidity until analysis at ages of 28 days and 18 months.

For fresh pastes, isothermal calorimetry measurements were carried out using a TA Instruments TAM Air isothermal calorimeter at 25 ± 0.02 °C. The materials were mixed externally, then 20 g samples of paste were weighed into plastic ampoules and placed into the calorimeter. Heat output was measured for 14 days (336 hours).

After curing, the samples were crushed, ground in an agate mortar, and sieved to <63 μm prior to analysis. X-ray diffraction (XRD) was undertaken using a STOE STADI P diffractometer (Cu Kα radiation, 1.5418 Å) with an imaging plate detector (IP-PSD) to collect data from 10° < 2*θ* ≤ 60°, and angle-corrected using an external silicon standard. Fourier transform infrared (FTIR) spectroscopy was undertaken on a Perkin Elmer Spectrum 2000 spectrometer in mid-infrared (MIR) mode, using 2 wt% of powdered sample in a pressed KBr disc. Solid-state ^29^Si MAS nuclear magnetic resonance (NMR) spectra were collected on a Varian VNMRS 400 (9.4 T) at 79.438 MHz using a 6 mm zirconia rotor, at a 6 kHz spinning rate. Pulse duration was 4.5 μs (90°), with a 10 s relaxation time for a minimum of 6000 repetitions, with chemical shifts externally referenced to tetramethylsilane (TMS) at 0 ppm. ^27^Al MAS NMR spectra were collected on a Varian VNMRS 400 (9.4 T) at 104.199 MHz using a 4 mm zirconia rotor, at a 14 kHz spin rate. Pulse duration was 1 μs (25°), with a 0.2 s relaxation time for a minimum of 5200 repetitions. Chemicals shifts were referenced externally to 1 M aqueous Al(NO_3_)_3_.

Scanning electron microscopy (SEM) was carried out on monolithic samples sliced using a diamond wafering blade; each specimen was mounted in epoxy resin, ground and polished using diamond paste to a 1 μm finish. Analysis was undertaken on a Hitachi TM3030 desktop SEM at 15 kV accelerating voltage, operating under a low vacuum, so coating of the samples was not needed. This was coupled with energy dispersive X-ray analysis (EDX) using a Bruker Quantax 70 Energy Dispersive X-ray Spectrophotometer for elemental mapping and spot analysis.

All samples analysed by thermogravimetry (TGA) were flushed with nitrogen for 30 minutes prior to analysis to remove surface water. Samples cured for 28 days were analysed using a Perkin Elmer Pyris 1 TGA, using 20 mg of material in an alumina crucible, heating at 10 °C min^−1^ up to 1000 °C in a nitrogen atmosphere. Aged (18 months) samples were analysed in a Perkin Elmer TGA 4000 instrument, using 20 mg of material in an alumina crucible, heating at 5 °C min^−1^ up to 950 °C in a nitrogen atmosphere. For these samples, the composition of the gases released during the TGA test was also determined using a Hiden Analytical mass spectrometer (MS) attached to the thermogravimeter.

## 3. Results and discussion

### 3.1. Early age evolution of a Mg(OH)_2_-modified sodium carbonate-activated slag cement

**Fig. 1:**
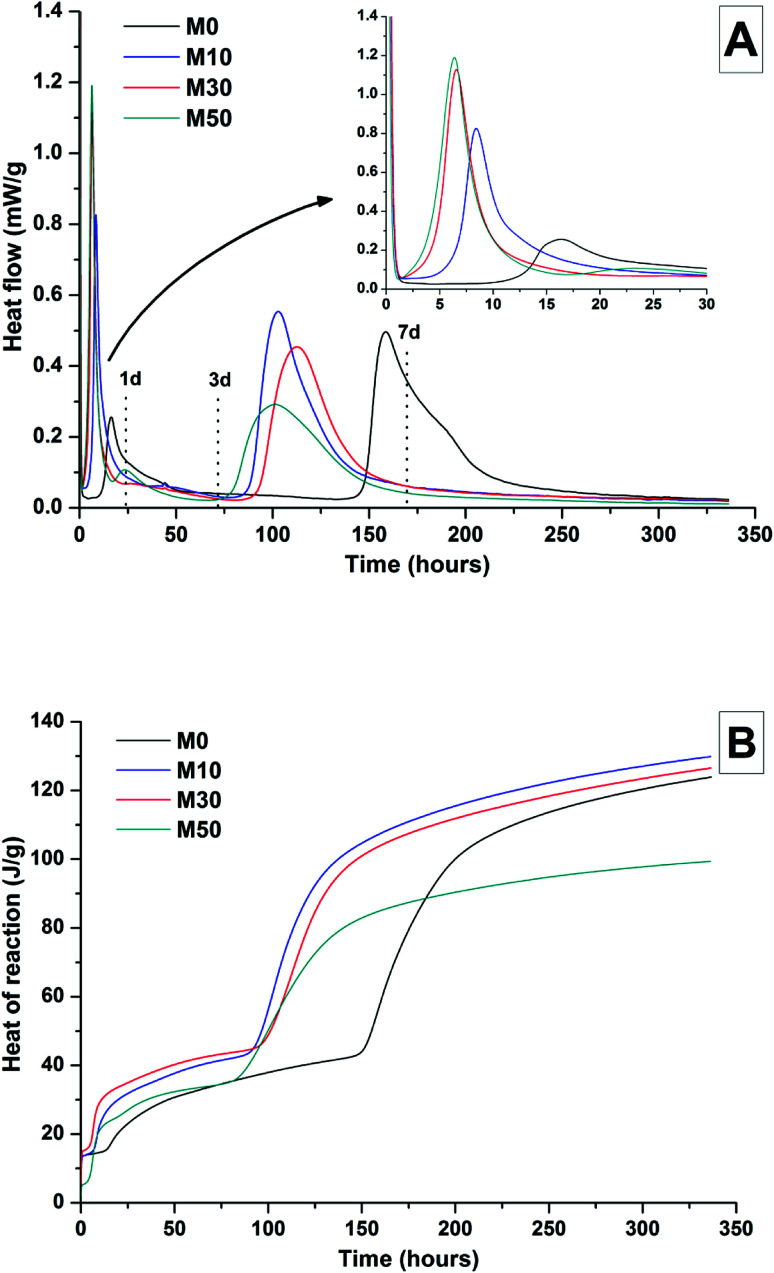
Isothermal calorimetry curves (A) and cumulative heat of reaction (B) of a sodium carbonate activated slag cement, normalised to total sample mass, as a function of the Mg(OH)_2_ content (percentage replacement of GGBS by Mg(OH)_2_ as marked in legend).

**Fig. 2:**
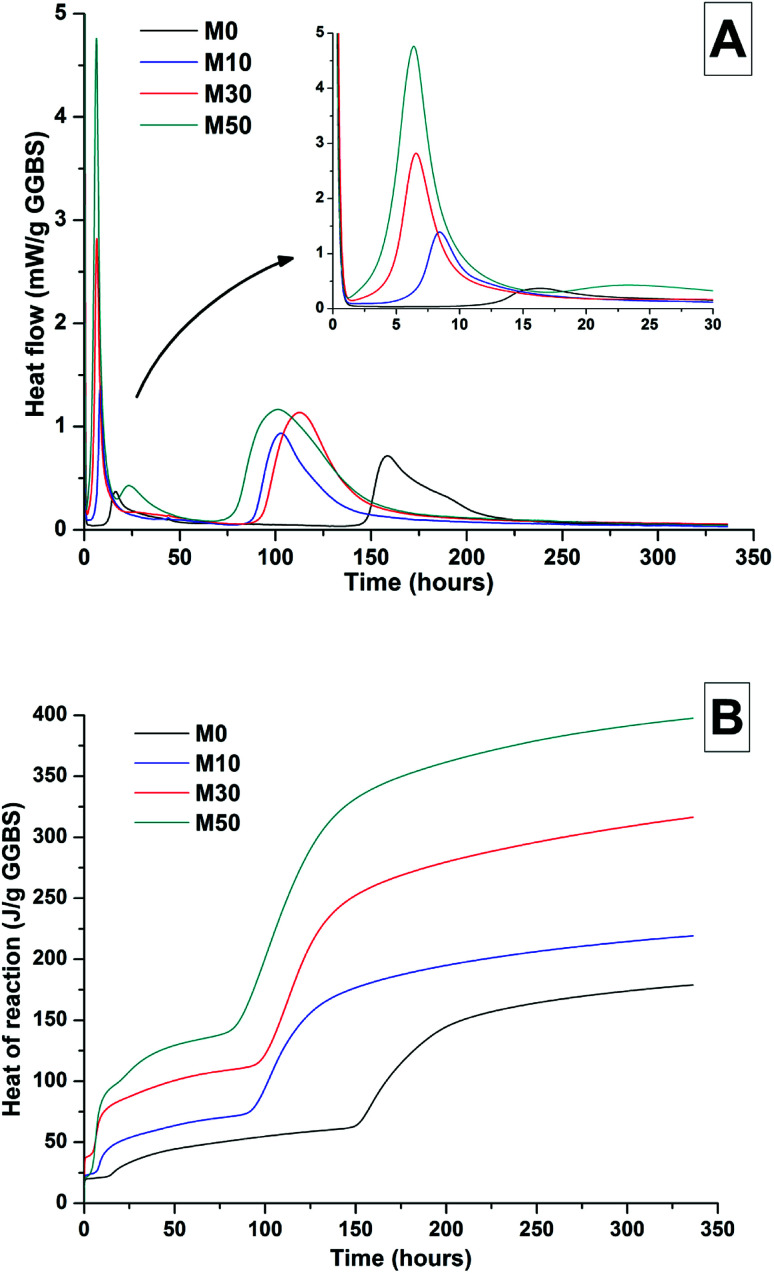
Isothermal calorimetry curves (A) and cumulative heat of reaction (B) of a sodium carbonate activated slag cement, normalised to mass of GGBS, as a function of the Mg(OH)_2_ content (percentage replacement of GGBS by Mg(OH)_2_ as marked in legend).

**Fig. 3:**
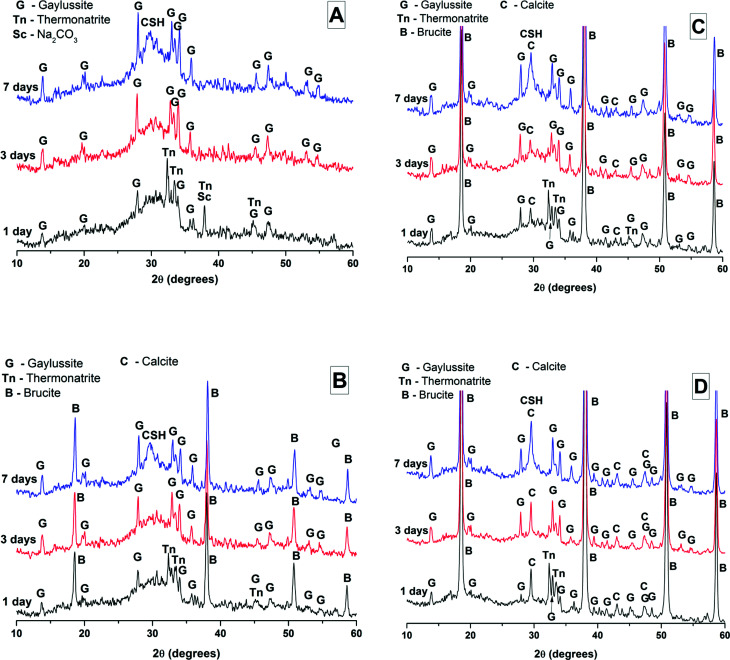
X-ray diffractograms of sodium carbonate activated slag cements after 1, 3 and 7 days of curing, containing (A) 0 wt%, (B) 10 wt%, (C) 30 wt% and (D) 50 wt% replacement of GGBS by Mg(OH)_2_. In all graphics, “CSH” indicates the diffuse reflection of calcium silicate hydrate-type gels, in this case substituted by both Na and Al.

The effects of GGBS replacement by Mg(OH)_2_ on the reaction kinetics of a sodium carbonate-activated slag cement were determined by isothermal calorimetry, Fig. 1 and 2, along with XRD analysis (Fig. 3) of the pastes during the first week of curing. Calorimetric data are normalised to both overall mass of sample (Fig. 1), and to content of dry GGBS (Fig. 2) for further interpretation.

Three main regions are identified from the calorimetric curves: <1 h, <24 h and 3–7 days, that correspond to distinct exotherms. Within the first hour, initial particle wetting and dissolution, and disturbance from the insertion of vials into the calorimeter, dominate. Following this, each sample exhibited an exotherm between 5-24 h, which can be attributed to formation of gaylussite (Na_2_Ca(CO_3_)_2_·5H_2_O, powder diffraction file (PDF) # 074-1235) and thermonatrite (Na_2_CO_3_·H_2_O, PDF #008-0448) identified by XRD in all the samples after 1 day of curing (Fig. 3), independent of the content of Mg(OH)_2_. Additionally, calcite was identified in samples with higher contents of Mg(OH)_2_ (Fig. 3C and D). These results are consistent with what has been reported for sodium carbonate activated slag cements, where the Ca^2+^ released from the dissolving slag reacts with CO_3_^2−^ from the activator to form carbonate salts such as calcite and gaylussite, increasing the pH through the release of hydroxide ions.^4,6^

The exothermic peak associated with the formation of carbonate phases was significantly delayed in cements without Mg(OH)_2_, appearing ∼10 hours later than for samples with 30 wt% and 50 wt% Mg(OH)_2_. After this event, negligible heat evolution was observed for up to 3 days in all cases, although the XRD results (Fig. 3) demonstrated that the formation of gaylussite at the expense of thermonatrite continued during this time.

Between 3 and 7 days each sample exhibited a further large exotherm, consistent with an acceleration-deceleration period, associated with the nucleation, growth and precipitation of reaction products including a calcium silicate hydrate (C-S-H)-type gel, as evidenced by the appearance of a diffuse scattering feature at ∼29° 2*θ* in the X-ray diffractograms of all the cements assessed (Fig. 3). The formation of this gel – which is likely to contain significant substitution by alkalis and aluminium, and so is more accurately described as C-(N-)A-S-H^28–30^ – was significantly accelerated in each of the samples containing Mg(OH)_2_.

The overall heat output of 10 wt% and 30 wt% substitution of Mg(OH)_2_ (M10 and M30 in Fig. 1) is similar to that without Mg(OH)_2_ addition (M0). This demonstrates that Mg(OH)_2_ can be safely added to an alkali-carbonate activated slag matrix without prompting an increased exotherm, which is highly desirable in the context of nuclear waste immobilisation. The heat of reaction is spread over a much wider timeframe than is typical in Portland cement blends, where the majority of heat is evolved within 40 h.^31,32^ This brings the opportunity to lower the wasteform peak temperature and ensure safer processing. Further replacement of GGBS by Mg(OH)_2_, up to 50 wt% (M50), lowers the overall heat of reaction due to the reduced quantities of GGBS available to react, and the increased quantity of water within this sample diluting the heat output per gram of total material (as Fig. 1 is normalised to total sample mass).

Normalising the data to the mass of dry GGBS (Fig. 2B) highlights a clear progression towards higher heat of reaction per gram of GGBS as the Mg(OH)_2_ content is increased up to 50 wt%, indicating either increased dissolution of the GGBS or a chemical reaction involving Mg(OH)_2_; the latter possibility will be the particular focus of the following sections of this paper. Presentation of the heat flow data normalised in this way (Fig. 2A) also shows the monotonic increase in the intensity of the first heat release peak (up to 15 hours) with Mg(OH)_2_ addition. Relative to the other samples, the composition containing 50 wt% Mg(OH)_2_ produces more heat per gram of GGBS during the timeframe associated with C-(N)-A-S-H formation (3–7 days), suggesting that there may be more extensive dissolution of the slag in this sample.

The effect of Mg(OH)_2_ addition in accelerating the precipitation of bulk reaction products (*i.e.* earlier onset of the second main reaction peak in all samples containing Mg(OH)_2_ compared to the M0 sample in Fig. 1 and 2) could be a consequence of either a threshold effect of increasing the Na_2_O/GGBS ratio upon Mg(OH)_2_ incorporation (see Section 2.2 and Table 2), or a filler effect from the Mg(OH)_2_ itself. The higher proportion of activator to GGBS might have moved the composition beyond a point at which the formation of hydration products is accelerated, compared to the baseline mix without Mg(OH)_2_.

However, as the absolute quantity of Na_2_CO_3_ is kept constant, and the higher water content will serve to dilute its alkalinity, the acceleration of reaction product precipitation might also be linked to a filler effect, whereby the added particles of Mg(OH)_2_ act as sites around which reaction products can nucleate and precipitate.^33,34^

According to the data in Fig. 2, the accelerating effect does not increase with higher additions of Mg(OH)_2_ beyond 10 wt%, which tends to suggest that nucleation rather than a threshold chemical ratio is the controlling factor.

### 3.2. Long term structural evolution of a Mg(OH)_2_ modified sodium carbonate activated slag cement

#### 3.2.1. X-ray diffraction

**Fig. 4:**
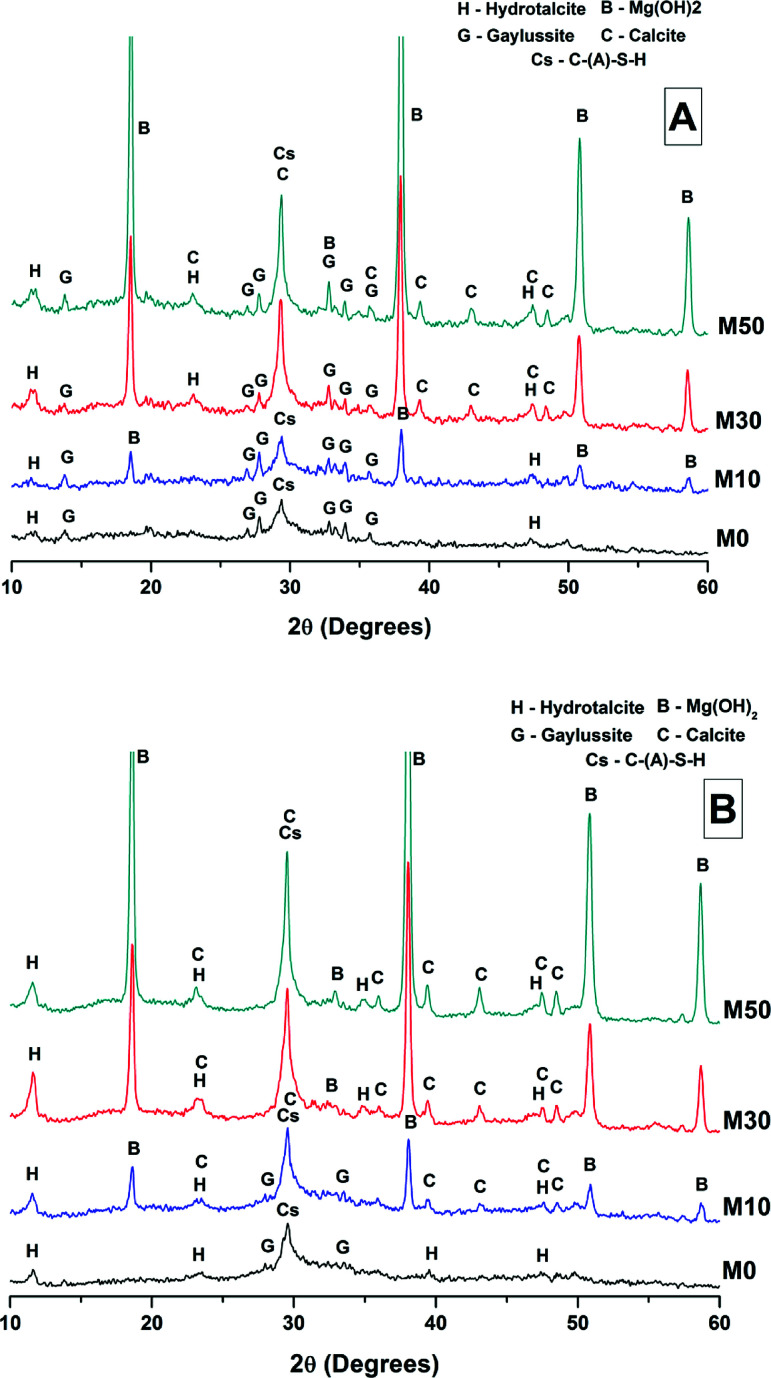
X-ray diffraction patterns of sodium carbonate activated slag binders after (A) 28 days, and (B) 18 months of curing. The strongest calcite reflections in sample M50 are truncated for visual clarity in presentation.

After 28 days of curing, X-ray diffraction patterns (Fig. 4A) show the continued growth of a C-S-H type phase when compared with the 7 days data in Fig. 3. High intensity reflections of the crystalline phases brucite (Mg(OH)_2_) and gaylussite were also identified. In all of the samples a reflection at 11.6° 2*θ* was identified, and assigned to the formation of a hydrotalcite-like phase resembling Mg_6_Al_2_(CO_3_)(OH)_16_·4H_2_O (PDF # 041-1428) but potentially with differences in Mg/Al ratio and/or interlayer species; the effects of these two factors are convoluted in the XRD analysis of hydrotalcite-group phases relevant to alkali-activated cements.^35^ This phase was not observable after 7 days of curing (Fig. 3), and so has grown in quantity and/or crystallinity during ongoing curing of the hardened samples. The main reflection assigned to this phase is especially prominent in samples with high contents of Mg(OH)_2_ (Fig. 4A, M30 and M50). More intense reflections assigned to calcite (CaCO_3_, PDF # 005-0586) and brucite were observed in the specimens containing 30 wt% and 50 wt% Mg(OH)_2_. The most intense reflection of calcite at 29.4° 2*θ* overlaps with the broad C-(N-)A-S-H reflection, however secondary reflections at 39.4°, 43.1°, 47.5° and 48.5° 2*θ* confirm the presence of large quantities of calcite in both of these samples.

Magnesium is known to be incorporated into calcite, forming magnesian calcites. This has been well studied within marine calcites, with Mg incorporation varying depending on *p*CO_2_, temperature and pH.^36,37^ However, any possible formation of magnesian calcite would be difficult to determine from the XRD data presented here; although magnesium incorporation affects the calcite lattice, resulting in a slight shift in the diffraction peaks, at low levels of incorporation this is difficult to discern from diffractograms as complex as those shown in Fig. 4. In the literature higher Mg : Ca ratios are observed to affect CaCO_3_ polymorph formations and stabilities, stabilising aragonite and vaterite by retarding growth of calcite, and enhancing the formation of monohydrocalcite.^37–40^ None of these are identified within any of the samples analysed here, suggesting that there is little involvement of the added Mg(OH)_2_ in such processes, or that the quantities formed are too low (or of low crystallinity) to be easily detected in this XRD analysis.

Although all of the cements analysed here show similar phases after 28 days of curing, their compressive strengths differ notably (Table 3); lower compressive strengths were obtained as more Mg(OH)_2_ replaces GGBS. In the mix designs used here (Table 2), Mg(OH)_2_ addition also necessitated an increased water to solids ratio to enable mixing and casting. In alkali activated materials this increased water content would typically reduce the concentration of the activator, however this does not occur here as activator dose was held constant with respect to the total solids content, meaning that the Na_2_O/GGBS ratio increases at higher Mg(OH)_2_ contents. The fact that the sample without Mg(OH)_2_ displays a lower strength than with 10 wt%, despite its lower water/solids ratio, suggests that the addition of Mg(OH)_2_ does influence the bulk properties of the cements in a substantive way.

**Table 3:** Compressive strengths of the sodium carbonate activated slag cements after 28 days of curing. Tests were undertaken in triplicate on 50 mm paste cubes

	M0	M10	M30	M50
Compressive strength (MPa)	49.8	55.6	40.8	20.8
Standard deviation	4.2	3.3	1.1	0.2

The phase assemblage of each binder continued to develop from 28 days up to 18 months of curing (Fig. 4B), with only minor gaylussite reflections identified in the samples at 18 months. In place of gaylussite, the continued formation of calcite was observed in all the samples, increasing in intensity with higher degrees of replacement of the slag by Mg(OH)_2_. A high intensity reflection of hydrotalcite was observed in all samples, displaying especially strong reflections in the sample with 30 wt% Mg(OH)_2_ (Fig. 4B, M30). The reflections assigned to Mg(OH)_2_ were strong in all samples in which Mg(OH)_2_ was added, suggesting that if there was any chemical reaction between Mg(OH)_2_ and the other mix constituents, it was far from complete. Thermodynamic calculations of the stable phase assemblage for sodium carbonate-activated slag binders containing high Mg contents have predicted brucite as a stable constituent of the phase assemblage,^41^ and the observation of its limited reaction here is consistent with those calculations. The increasing content of Mg(OH)_2_ did not appear to influence the observed phase assemblage, other than increasing the intensity of the reflections assigned to calcite, C-(N-)A-S-H and hydrotalcite, which is commensurate with an increased degree of slag dissolution due to the increased activator/slag ratio in the higher-Mg(OH)_2_ samples.

The overall phase evolution of these slag cements was similar to the processes previously identified in sodium carbonate activated slags.^4,42^ The major difference from the established literature is the presence of calcite, and the absence of the CaCO_3_ polymorphs aragonite and vaterite, which is contrary to other results available in the literature for aged samples.^4,42^

For sodium carbonate activation of a slag with a comparable composition to that which was studied here, Myers *et al.*^30,41^ predicted the formation of a C-(N-)-A-S-H gel, calcite, the zeolitic phase natrolite (Na_2_Al_2_Si_3_O_10_·2H_2_O), a hydrotalcite-group Mg-Al LDH phase with OH^−^ groups dominating its interlayer (MA-OH-LDH), and a calcium monocarboaluminate phase in the AFm family (C_4_AĈH_11_). In this study, neither natrolite nor AFm phases were detected directly by XRD, however the presence of C_4_AĈH_11_ remains possible as its key Bragg reflections (PDF # 041-0219;^43^) present significant overlap with those of hydrotalcite-like phases, making confident identification difficult. The distinction between the MA-OH-LDH phase and carbonate-containing hydrotalcite-group phases is also challenging due to their similar diffraction patterns; this will be revisited below using thermogravimetry and mass spectroscopy. Gaylussite is not predicted to be a stable phase within these cements, which is in line with the decrease in its reflections here from 28 days to 18 months, and in agreement with other studies.^4^

#### 3.2.2. Fourier transform infrared (FTIR) spectroscopy

**Fig. 5:**
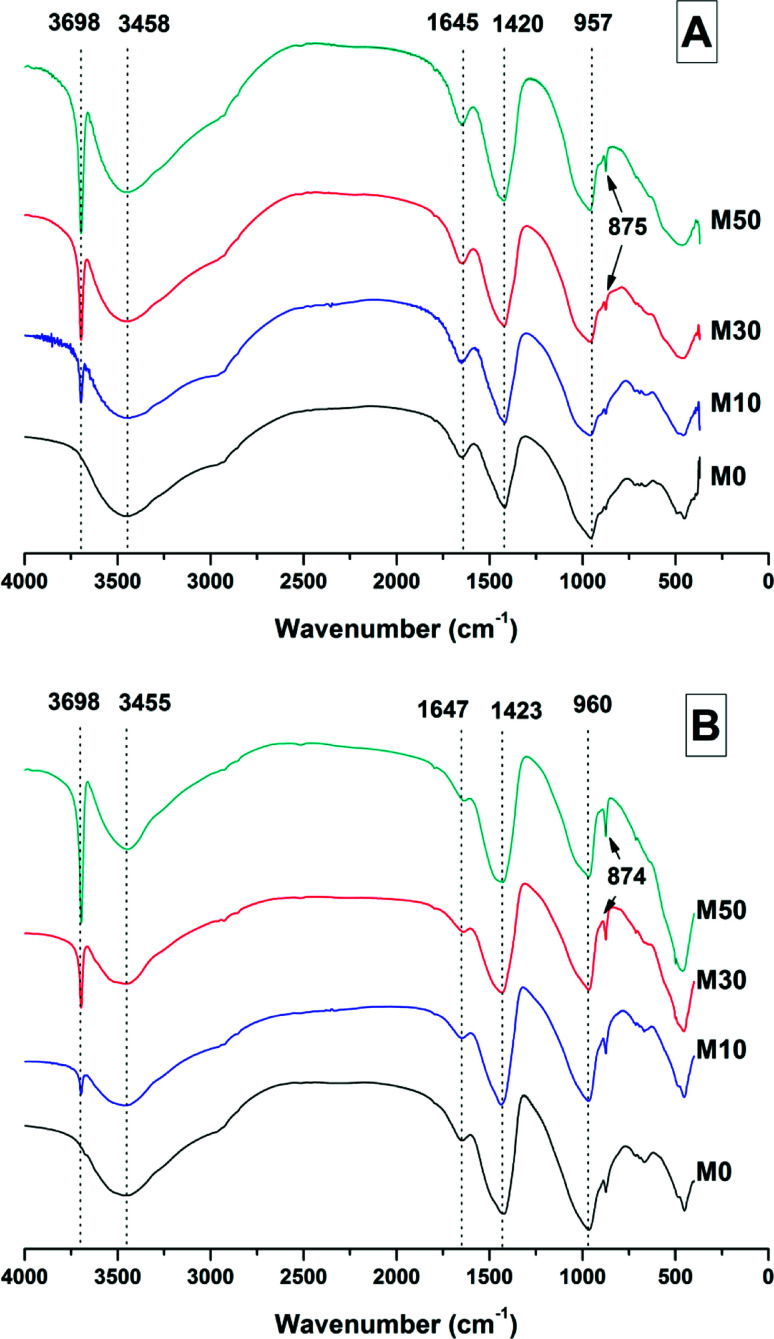
FTIR spectra of sodium carbonate activated slag binders after (A) 28 days, and (B) 18 months of curing.

Fig. 5 shows the infrared spectra of the samples at 28 days and 18 months. In both Fig. 5A (samples cured for 28 days) and Fig. 5B (samples cured for 180 days), the most notable difference among the samples is related to the sharp peak at 3698 cm^−1^, which is assigned to the O–H stretching vibration mode of brucite.^44^ As expected, reduced contents of Mg(OH)_2_ in the cement give a lower intensity in this band. Although significant quantities of Mg(OH)_2_ remain in the samples after 18 months of curing, especially when adding high contents of Mg(OH)_2_ (Fig. 5B, M50), a slight reduction in the intensity of this band is observed when comparing pastes containing lower Mg(OH)_2_ contents cured for 18 months, against the data at 28 days. This tentatively suggests Mg(OH)_2_ might not be completely inert, though there is potential for this to be an artifact of sample preparation or analysis, due to the 17 months sampling interval between data sets.

The broad band centred at 3458 cm^−1^ in each of the samples at 28 days, and 3455 cm^−1^ at 18 months, corresponds to the H–OH stretching mode of bound water, while the resonance at 1644 cm^−1^ is assigned to the bending mode of the H–OH bond.^45,46^ These resonances are typical of bound and interlayer water within C-A-S-H type gels,^47,48^ along with crystal water within a hydrotalcite-type phase and in gaylussite,^46^ consistent with the phases observed by X-ray diffraction (Fig. 4).

The Si–O–(Al,Si) stretching vibration mode of the C-(N-)A-S-H gel is identified at 957–960 cm^−1^,^47^ overlapping resonances from residual slag. The position of this band was not affected by the addition of different quantities of Mg(OH)_2_, which indicates that there were not significant changes identifiable in either the degree of crosslinking or the chemical composition of the C-(N-)A-S-H phase as a result of Mg(OH)_2_ addition.

The broad band at 1420 cm^−1^ is attributed to the ν_3_ carbonate stretching band typical of carbonate phases, consistent with the identification of calcite, gaylussite and the hydrotalcite-like phase in the XRD data (Fig. 3 and 4) for these samples.^46,49^ Carbonate vibrations from calcite were more clearly evident in a sharp peak (ν_2_ band) at 874–875 cm^−1^, which increased in intensity in the samples with higher contents of Mg(OH)_2_ and also with curing age. This correlates well with the trends in the XRD reflections assigned to calcite (Fig. 4).

**Fig. 6:**
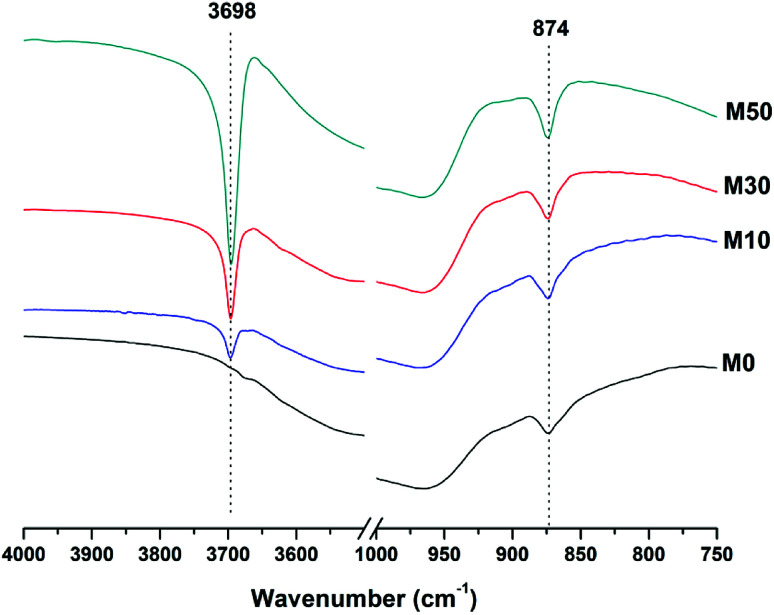
Expanded FTIR spectra of sodium carbonate activated slag binders after 18 months of curing.

Both the OH stretching (3800–3600 cm^−1^) and carbonate vibration (900–850 cm^−1^) regions of the FTIR spectra of aged samples are shown in an expanded view in Fig. 6, as these regions provide evidence for the formation (or absence) of other carbonate or hydroxy-carbonate phases.

Due to the large quantity of Mg(OH)_2_ in these samples, there might exist opportunities for the formation of magnesium carbonate phases. Magnesite (MgCO_3_) typically exhibits a sharp vibration at ∼885 cm^−1^,^46^ while other hydrated magnesium carbonates display sharp vibrations between 3700-3500 cm^−1^ (*e.g.* nesquehonite (MgCO_3_·3H_2_O) at 3570 cm^−1^,^46^ hydromagnesite (Mg_5_(CO_3_)_4_(OH)_2_·4H_2_O) at 3645, 3505 and 3445 cm^−1^,^46^ and dypingite (Mg_5_(CO_3_)_4_(OH)_2_·5H_2_O) at 3648 cm^−1 ^^50^). The absence of these vibration modes, combined with the lack of reflections characteristic of magnesium carbonates from the XRD data as shown above, rules out the formation of pure magnesium carbonates other than the hydrotalcite-like phase. This analysis also rules out the formation of the CaCO_3_ polymorphs aragonite and vaterite, as they display characteristic infrared vibrations in the region 900–850 cm^−1^.^46,51^

The possibility of magnesium incorporation within Ca-containing carbonate phases is somewhat more difficult to determine within these samples. Huntite (CaMg_3_(CO_3_)_4_) can be excluded, as this mineral exhibits strong vibrational modes at 892 and 869 cm^−1^,^46^ which are not observed in Fig. 6. Dolomite (CaMg(CO_3_)_2_) is known to have slow crystallisation kinetics so is rarely formed in cements in any case,^52^ and typically displays a sharp vibration at 881 cm^−1^.^53^ Incorporation of Mg into calcite to form a magnesian calcite is much more difficult to ascertain, as was the case for the XRD analysis in the preceding section. FTIR analysis of calcites of different Mg contents has shown that the wavenumber of the carbonate ν_2_ vibrational mode increases with the proportion of magnesium in the calcite structure, though this is typically only up to ∼876–7 cm^−1^ in naturally occuring magnesian calcites (*e.g.* Ca_0.84_Mg_0.16_CO_3_ analysed in^54^). The carbonate ν_2_ vibration within these samples does not alter in position from 0 to 50% Mg(OH)_2_ addition, suggesting that there is little to no Mg incorporation within the calcite that is formed.

#### 3.2.3. Thermogravimetric analysis

**Fig. 7:**
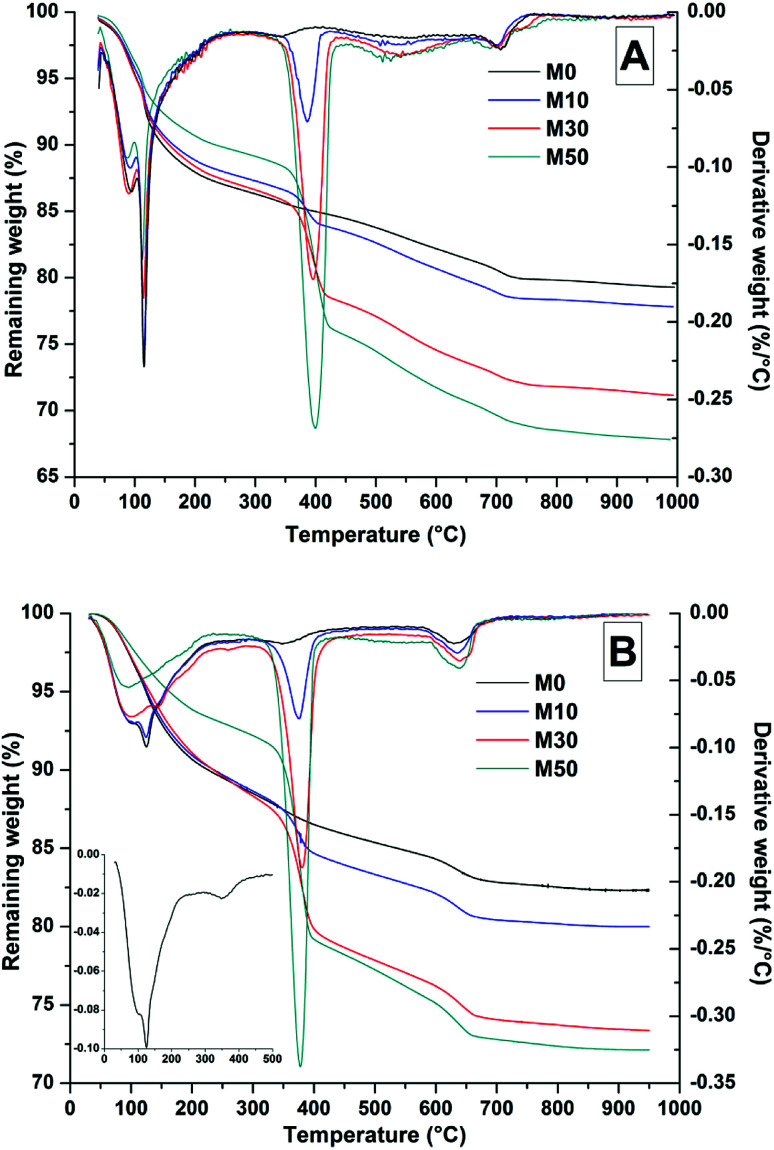
Thermogravimetric (TG) and differential thermogravimetric (DTG) analysis of samples after (A) 28 days curing and (B) 18 months curing, with M0 DTG data highlighted as an inset to show the characteristic hydrotalcite peaks.

**Fig. 8:**
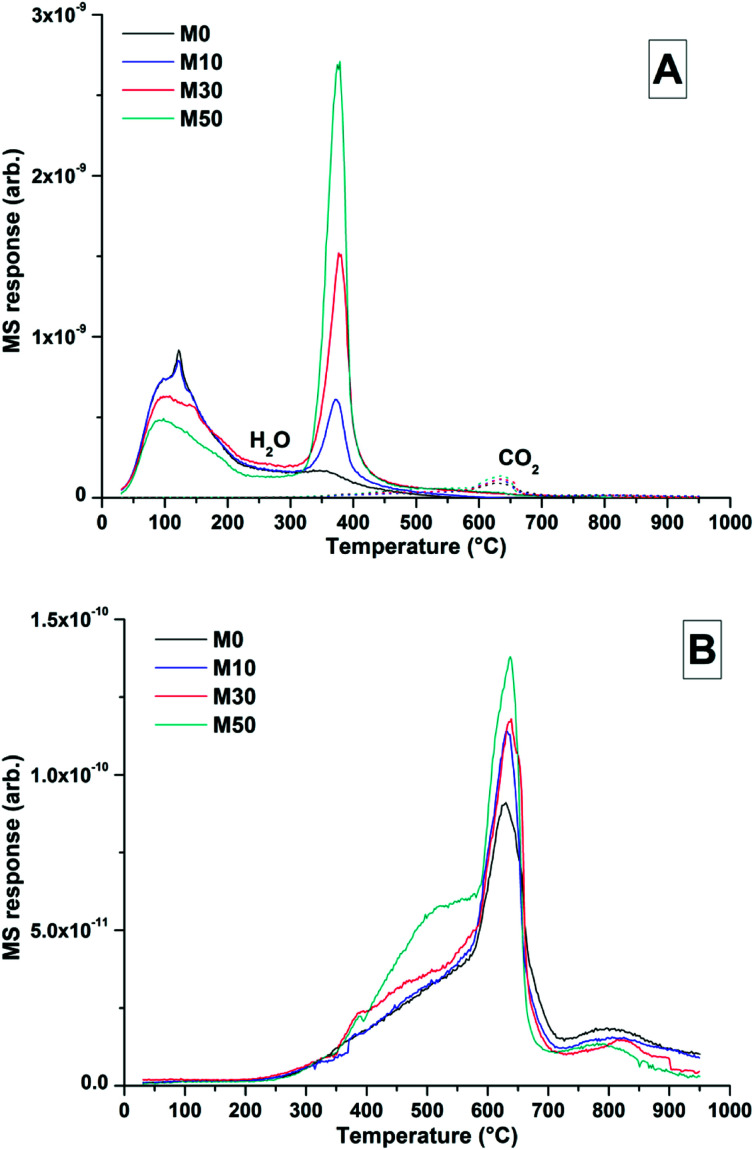
MS data for all samples after 18 months curing, (A) combined H_2_O and CO_2_, (B) CO_2_ response.

Thermal analysis of the samples after 28 days (Fig. 7A) and 18 months (Fig. 7B and 8) shows a mass loss at temperatures below 200 °C, which can be attributed to removal of loosely bonded water, and the initial loss of the interlayer and structural water present in the C-(N-)A-S-H.^55,56^ The sharp peak at 110 °C is assigned to gaylussite dehydration,^57^ in agreement with the identification of this phase by XRD analysis, where notably reduced gaylussite intensities were observed after 18 months of curing.

Dehydroxylation of Mg(OH)_2_ was prominent at ∼400 °C, where mass loss increased commensurately with the addition of Mg(OH)_2_. At ∼630 °C, calcite decomposition was observed, followed by gaylussite decarbonation.^58^ The decomposition of the hydrotalcite-like phase was more difficult to determine as it typically exhibits a multi-step process of dehydration, dehydroxylation and/or decarbonation up to ∼600 °C, depending on the structure and interlayer anions,^59–61^ and potentially overlapping with the mass loss due to Mg(OH)_2_ decomposition. This is highlighted within the differential thermogram of the 18 months cured sample without Mg(OH)_2_ addition (inset Fig. 7B), which shows a distinct shoulder at ∼200 °C and further mass loss at ∼350 °C. This is characteristic of a hydrotalcite-like phase^62^ and can be noted in the other 18 months samples in Fig. 7B, where there is an apparent shift in the Mg(OH)_2_ decomposition peak from ∼380 °C towards a lower temperature, ∼350 °C.

The TG-MS data (Fig. 8) for samples with different contents of Mg(OH)_2_ addition after 18 months might provide further insight into the nature of this Mg-LDH phase, as the XRD analysis presented above was not able to unequivocally define whether it is a solely hydroxide-containing product, or whether it contains carbonate. In these samples, the mass loss at ∼350 °C only registered in the mass spectroscopy data as H_2_O, not as CO_2_. Typically a carbonated hydrotalcite-like phase would evolve interlayer CO_2_ in this temperature range.^63^ Plotting data for CO_2_ alone (Fig. 8B) does reveal slight evolution of CO_2_ here, however this is part of a broader decarbonation culminating in calcite decomposition at ∼630 °C rather than a discrete event which could be attributed to a carbonate-containing LDH. Unfortunately Mg-Al-CO_3_ LDH phases have been shown to release CO_2_ at >400 °C,^61^ which overlaps with CaCO_3_ decomposition. Although evidence from thermodynamic modelling predictions of Myers *et al.*^30,41^ suggest that the hydrotalcite-group phase formed in these systems is in fact an MA-OH-LDH phase rather than a carbonate containing phase, TG-MS is unable to definitely determine this within these samples.

#### 3.2.4. Microstructural features of aged Mg(OH)_2_-containing sodium carbonate-activated cements

**Fig. 9:**
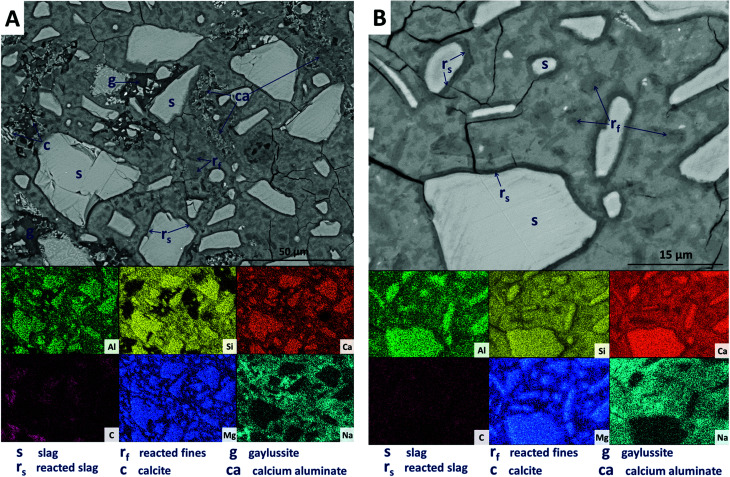
Backscattered electron micrographs ((A) low magnification; (B) higher magnification) and corresponding EDX maps of a sodium carbonate activated slag without Mg(OH)_2_ addition, after 18 months of curing.

Backscattered electron (BSE) micrographs of the sample without Mg(OH)_2_ (M0) are displayed in Fig. 9, with corresponding elemental maps. The slag grains (labelled ‘s’ in the micrographs) have undergone varying degrees of dissolution, resulting in the formation of a dense matrix composed of a C-(N-)A-S-H type gel, as evidenced by the widespread distribution of Al, Si, Ca and Na, in which all the other features are embedded. The remnant slag grains are surrounded by thin darker reaction rims (labelled ‘r_s_’ in Fig. 9A), clearly observed at a higher magnification (Fig. 9B). This ‘rim’-like region is particularly evident for slag fines, where the slag grains have entirely reacted, leaving regions with darker greyscale values than the bulk matrix (these are labeled ‘r_f_’ in Fig. 9A and particularly evident in the Mg map in Fig. 9B), without a bright remnant slag core. This region is richer in Mg than the bulk matrix, indicating the presence of the hydrotalcite-like phase identified above, and consistent with observations by other authors in slag cements activated by sodium hydroxide or silicate,^64,65^ and in aged sodium carbonate-activated slag cements.^6^

Large darker regions (labelled as ‘g’ in Fig. 9A) are assigned to gaylussite, identified by enrichment in Na and C compared to the bulk matrix, and the absence of Al and Si. Similar features have been observed in sodium carbonate/silicate-activated slag cements,^4^ where gaylussite is reported as secondary reaction product. Formation of calcite (labelled ‘c’ in Fig. 9A), corresponding to the regions observed in the EDX maps to be rich in Ca and C but lacking in Al and Si, was observed around gaylussite. The large darker patches (labelled ‘ca’ in Fig. 9A) are particularly low in Si but still contain appreciable quantities of Ca, C and Al, suggesting the formation of a hydrous calcium carboaluminate phase. This may potentially be the monocarbonate AFm (C_4_AĈH_11_, or ‘CO_2_-AFm’) phase predicted by Myers *et al.*^30,41^ in sodium carbonate-activated slag cements *via* thermodynamic modelling, and recently identified by Ke *et al.*^6^ in aged cements of that composition.

**Fig. 10:**
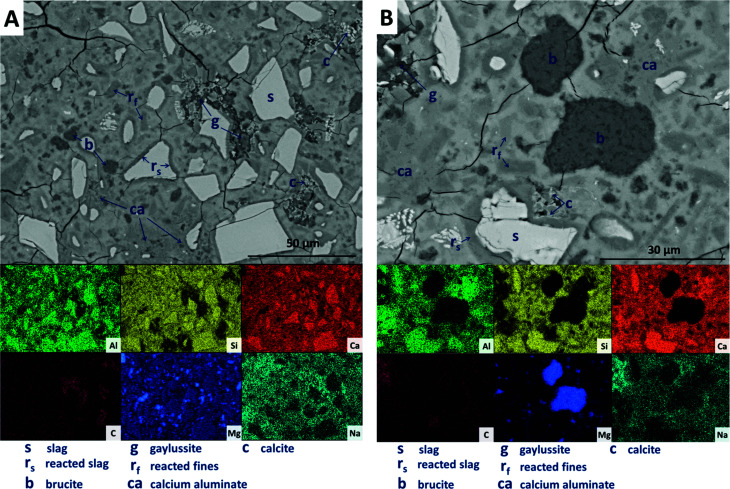
Backscattered electron micrographs ((A) low magnification; (B) higher magnification) and corresponding EDX maps of a sodium carbonate activated slag with 10 wt% Mg(OH)_2_ addition, after 18 months of curing.

SEM results for specimens with 10 wt% Mg(OH)_2_ (sample M10) are shown in Fig. 10. Again, a significant quantity of unreacted slag (marked s) remained, around which reaction rims (r_s_) have developed. As in Fig. 9, remnant reaction rims from dissolution of slag fines (r_f_) are observed, along with the presence of gaylussite (g), and with calcite (c) forming on the edges of gaylussite patches. Small Mg(OH)_2_ particles (b) are observed throughout the sample, and do not appear to have undergone any reaction but instead remain embedded within the C-(N-)A-S-H rich bulk matrix. Fig. 10B presents a higher magnification view of two large Mg(OH)_2_ particles, which are embedded in the bulk gel. The EDX maps do not reveal any reaction region surrounding the particles, nor is there evidence of decalcification or Al enrichment in the bulk surrounding these particles which would indicate MA-OH-LDH formation around the Mg(OH)_2_ particles. This suggests that the Mg(OH)_2_ is not participating in any reaction with the slag, and is merely acting as a filler or site for nucleation for a C-(N-)A-S-H gel.

**Fig. 11:**
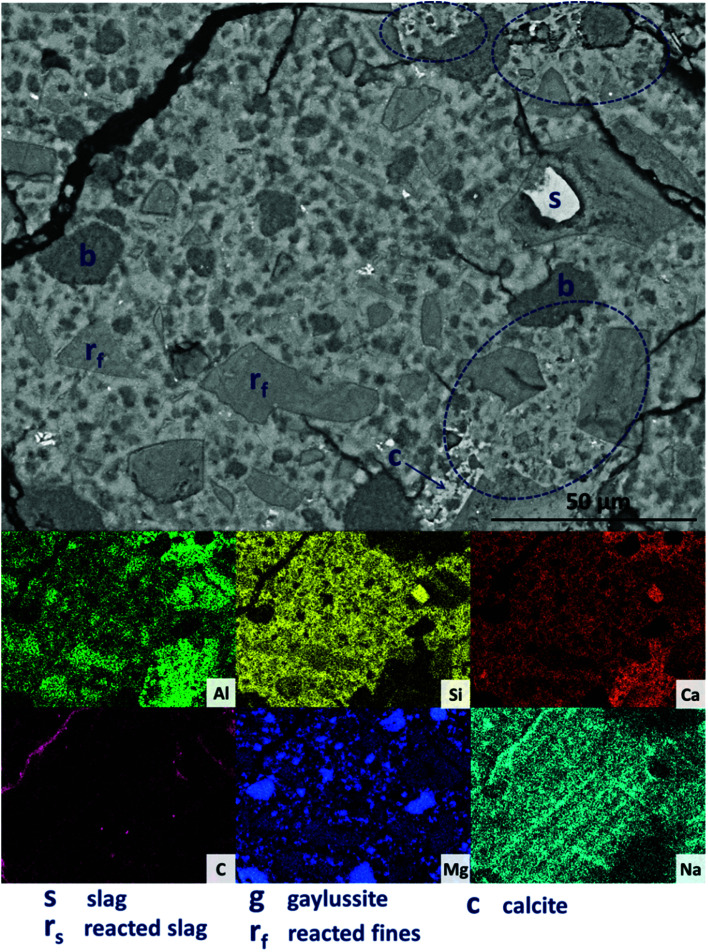
Backscattered electron micrograph and corresponding EDX maps of a sodium carbonate activated slag with 30 wt% Mg(OH)_2_ addition, after 18 months of curing.

Further increasing the level of replacement of GGBS by Mg(OH)_2_ (30 wt%, sample M30) resulted in a substantial change in the microstructure of the hardened cement paste, and an increased tendency to cracking and desiccation during sample preparation for electron microscopy. In M30 (Fig. 11), little unreacted slag remains, and that which is left is surrounded by large reaction rims (r_s_), which are higher in Mg and Al than the bulk matrix. Significant amounts of finely dispersed Mg(OH)_2_ particles (b) are observed embedded throughout the matrix. As in Fig. 10, these particles appear to be unreacted, with no transition or visible reaction rims that could indicate participation in the chemical reaction of the cementing system. The XRD analysis (Fig. 4B) suggested the presence of increased amounts of calcite in this sample compared with those with lower Mg(OH)_2_ contents. The observations here from SEM are consistent with this, as large clusters of calcite (c) are identified, instead of the gaylussite observed in the specimens with lower Mg(OH)_2_ contents. Like the gaylussite, calcite is largely confined to large clusters rather than being uniformly distributed throughout the sample. It is, however, also present as minor clusters associated with the regions identified above as potentially being the CO_2_-AFm phase, in Fig. 11. This micrograph shows the extensive formation of this hydrous calcium carboaluminate phase in three locations, highlighted by dashed regions and identifiable by a very strong content of Ca and Al within the EDX maps.

**Fig. 12:**
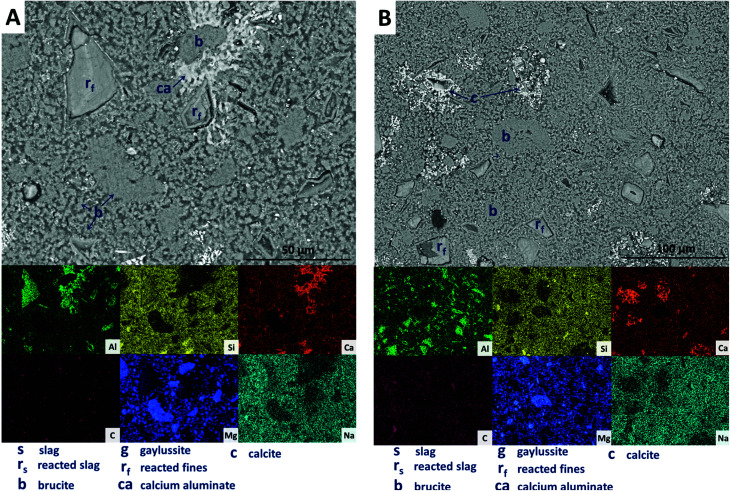
Backscattered electron micrographs (two different regions in A and B) and corresponding EDX maps of a sodium carbonate activated slag with 50 wt% Mg(OH)_2_ addition, after 18 months of curing.

The replacement of 50 wt% of the GGBS by Mg(OH)_2_ (M50) further changes the observed microstructure of the cement. No remnant slag grains are identified in Fig. 12A and B, having undergone full dissolution and leaving large reaction regions (r_f_) enriched in Mg and Al. These appear to have also developed a secondary reaction rim, deficient in Si and Ca relative to both the bulk and interior of the reaction regions. This was in some instances further encircled by a thin layer of calcite (c). Mg(OH)_2_ (b) was dispersed throughout the sample, both as large clumps and as smaller particles, commensurate with the very high replacement level in this sample. A distinct CO_2_-AFm growth (ca) can be observed in Fig. 12A, which appeared to be growing around one of the Mg(OH)_2_ particles, although no reaction region is identified within the particle itself, and Mg substitution into the AFm structure is not likely. The AFm phase appeared lighter in this micrograph than in previous samples, but this was likely a contrast effect due to the high Mg(OH)_2_ content and lack of unreacted slag, which typically appeared lighter in the micrographs. Looking at a wider section of the sample in Fig. 12B, the morphology was quite different from that of samples containing less Mg(OH)_2_. Very large clusters of calcite (c) were present, along with fine speckles of calcite distributed throughout the binder.

**Fig. 13:**
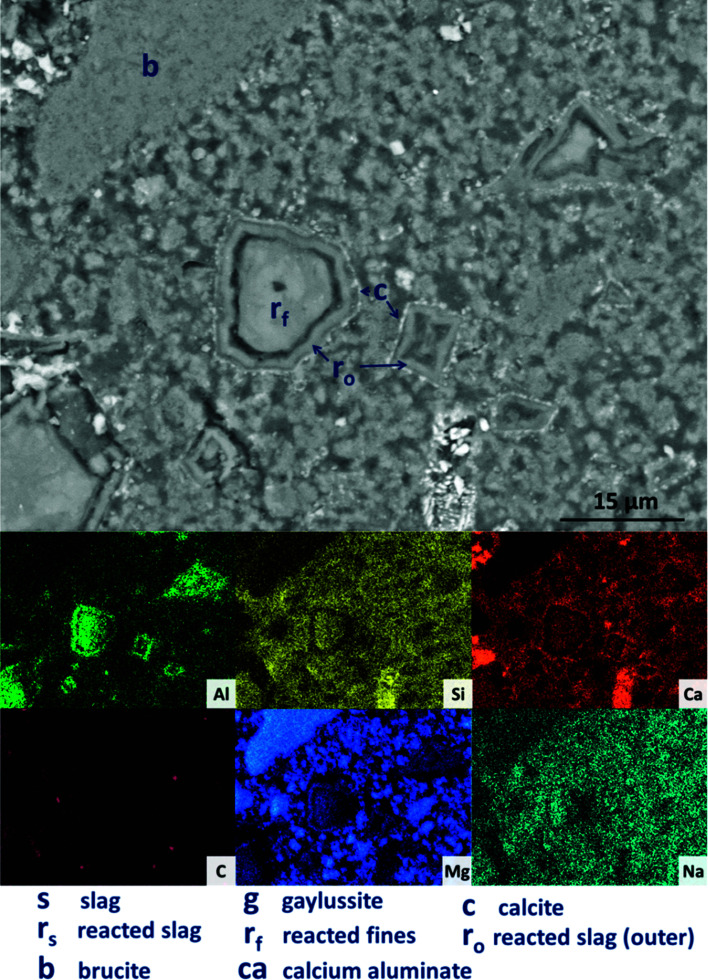
High magnification backscattered electron micrograph and corresponding EDX maps of a sodium carbonate activated slag with 50 wt% Mg(OH)_2_ addition, after 18 months of curing.

Looking more closely at one of the reacted slag particles in this sample, Fig. 13, distinct multiple reaction rims (r_o_) are observable. The inner reaction rim (r_f_) is similar in Si, Ca and Na concentrations to the bulk matrix, although enriched in Mg and Al. This is identified as formation of a C-(N-)-A-S-H gel intermixed with an MA-OH-LDH phase, as the degree of incorporation of Mg into C-S-H type gels is known to be very limited.^66^ The next reaction rim has a lower density darkened ring deficient in most elements (*i.e.* is a porous region), followed by another ring (r_0_) enriched in only Al and Mg, indicating the presence of the MA-OH-LDH phase alone. This was also observed in several reacted slag regions throughout the sample. Both sites highlighted also exhibit thin calcite (c) deposits around the outer rings; similar Liesegang-type ring formations have been observed in aged silicate-activated slag concrete,^67^ and their presence suggests that the reaction of these cements at advanced age, independent of the activator used, is likely to follow an Oswald supersaturation–nucleation–depletion cycle.

Further analysing the large Mg(OH)_2_ particle marked (b) in Fig. 13, the distributions of Ca, Si, Al and Na all reach up to the particle edge according to the EDX maps, indicating that this particle is embedded directly within a C-(N-)A-S-H gel. There is no preferential accumulation of Al along the particle edge which would indicate the formation of a hydrotalcite-like phase. Any formation of a magnesium silicate hydrate (M-S-H) as a reaction product would lead to competition between Ca-rich and Mg-rich phases for the Si, and this would leading to regions rich in Mg and Si only, to the exclusion of Ca. However, no such regions are identified along the edge of this Mg(OH)_2_ particle, nor any others in the analysis presented here, so it can be concluded that the bulk Mg(OH)_2_ did not react to form M-S-H to a significant extent within this system. This also indicates that the source of Mg for formation of the hydrotalcite-like phase identified by XRD analysis (Fig. 3) was the slag alone, which had an MgO content of 8.4 wt%, Table 1.

#### 3.2.5. Chemical composition of the main reaction products

Spot EDX measurements were taken throughout the bulk matrix, the reaction rims and the AFm phase. Table 4 details the average atomic ratios measured for these distinct features (rim, bulk, inner rim and outer rim) throughout all the samples after 18 months of curing. The Ca/Si ratio of the bulk gel remained fairly similar between samples M0 – M50, ranging from 1.09 to 1.22. This is a typical Ca/Si ratio for sodium carbonate activated slags,^4^ though higher than typically identified in sodium silicate activated binders.^68^ The same Ca/Si ratio was measured in the reaction rims featured in all of the samples studied, supporting the identification of these regions being intermixed MA-OH-LDH and C-(N-)A-S-H, while the bulk was largely a C-(N-)A-S-H gel with some MA-OH-LDH present.

**Table 4:** Average EDX spot map atomic ratios, and standard deviations associated with each, after 18 months of curing

		Ca/Si	Mg/Si	Ca/Al	Mg/Al	Al/Si
M0	Rim	1.13	0.47	2.36	0.95	0.49
	SD	0.18	0.10	0.54	0.11	0.06
	Bulk	1.09	0.17	3.50	0.53	0.31
	SD	0.15	0.05	0.62	0.11	0.03
M10	Rim	1.10	0.82	1.82	1.33	0.61
	SD	0.19	0.13	0.33	0.10	0.07
	Bulk	1.11	0.28	3.60	0.89	0.31
	SD	0.18	0.09	0.69	0.24	0.03
M30	Rim	1.12	1.47	1.48	1.92	0.77
	SD	0.20	0.15	0.31	0.10	0.07
	Bulk	1.09	0.47	4.07	1.74	0.27
	SD	0.17	0.15	0.83	0.57	0.02
M50	Inner rim (r_f_)	1.16	2.45	1.15	2.41	1.02
	SD	0.17	0.20	0.20	0.18	0.09
	Outer rim (r_o_)	1.19	4.95	0.73	2.88	1.74
	SD	0.24	1.19	0.21	0.23	0.49
	Bulk	1.22	2.22	4.21	7.69	0.29
	SD	0.22	0.52	0.93	2.11	0.03

The Al/Si values of the rims were consistently higher than those of the bulk (up to 1.74 for sample M50), which also correlated with a higher Mg/Al ratio in the rims. The exception to this is the measured Mg/Al ratio of 7.69 in M50, where it is postulated that the bulk spot maps included Mg(OH)_2_ particles due to their fine dispersal throughout the microstructure of this sample.

**Fig. 14:**
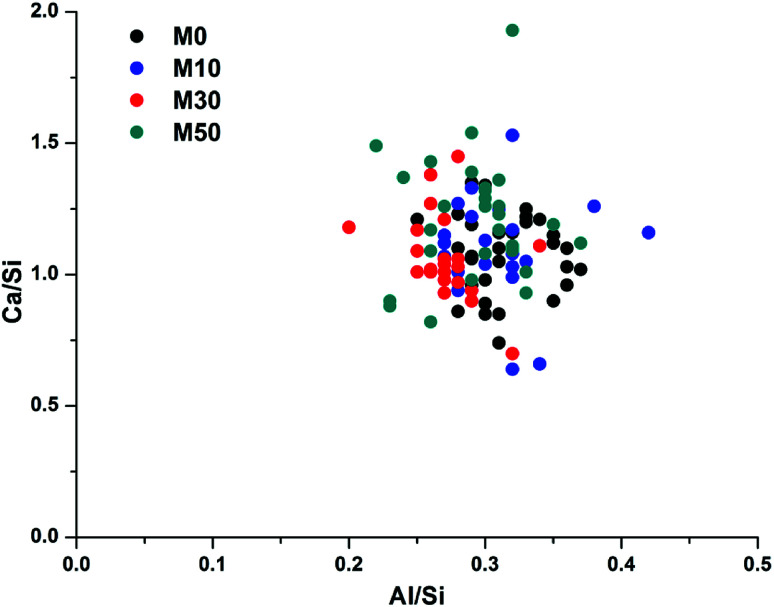
Plot of EDX spot map atomic ratios comparing Ca/Si *vs.* Al/Si in the bulk matrix.

Fig. 14 correlates Ca/Si *vs.* Al/Si atomic ratios of the bulk matrix in all of the samples. There is considerable scatter between the individual points, however there is no discernible difference between any of the samples, suggesting that the overall bulk composition of calcium, aluminium and silicon within the gel does not change as Mg(OH)_2_ is added into the system. This does not, however, mean that no changes are occurring; as changes in Al–Si ordering, or interactions with excess Na, are not easily observed through bulk EDX. There will be an increase in Na/Si ratios when 30 wt% or 50 wt% Mg(OH)_2_ replaces GGBS, though Na is easily lost in sample preparation and so its analysis by EDX is often unreliable. A higher Na content raises the possibility of increased charge compensation within a C-(N-)A-S-H gel, leading to structural changes, or the potential formation of a co-precipitated N-A-S-H gel. Neither of these would be easily determined *via* bulk EDX analysis, but the following section will address these points by NMR analysis.

The Al/Si values of the bulk gel for samples M0 – M50 were very similar, at 0.27–0.31. Although this is high for a C-(N-)A-S-H gel, the true Al/Si ratio of the gel will be lower once the Al present in MA-OH-LDH is removed from the analysis (as determined below). We postulate that the nature of this gel is likely to change as a result of the increased Na/Si ratio associated with higher Mg(OH)_2_ replacement levels, as the Na_2_O/GGBS ratio also increased. Due to the significant quantity of Na_2_CO_3_ used relative to the GGBS in formulations M30 and M50 (9.75 and 14.62% Na_2_O eq. respectively), there is the potential for the additional formation of an N-A-S-H gel, as will be assessed in more detail in the following sections.

**Fig. 15:**
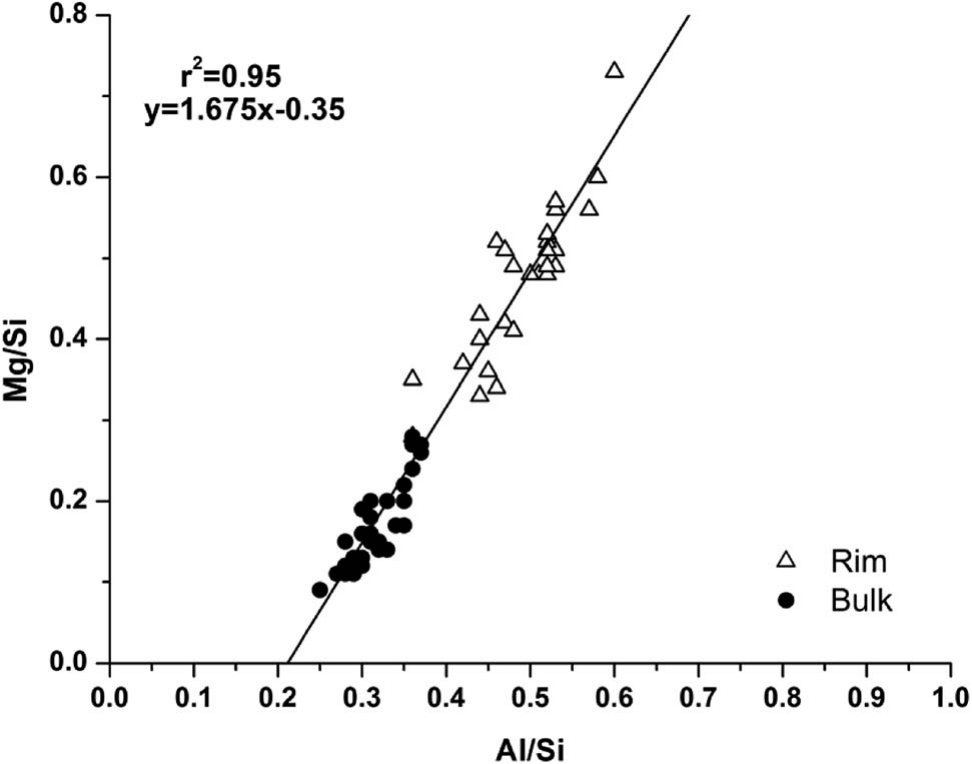
Plot of EDX spot map atomic ratios, comparing Mg/Si *vs.* Al/Si ratios of the 18 months cured sample without added Mg(OH)_2_ (M0).

Analysis of compositional changes within the bulk matrix is to some degree complicated by the formation of the MA-OH-LDH phase. Although this has been noted to mainly precipitate in rims surrounding GGBS particles (or in the remnants of dissolved particles), analysis of the Mg/Si *vs.* Al/Si spot map data (as plotted in Fig. 15 for M0) shows evidence for its formation within the bulk as well.

There is a clear positive linear correlation between Mg and Al contents, indicating LDH formation in this sample, in both bulk and rim regions. Linear regression of these data to zero Mg content (the intercept with the horizontal axis) shows that the Al/Si ratio in the C-(N-)A-S-H gel is 0.21, and the slope of the trendline indicates an Mg/Al ratio within the MA-OH-LDH phase of 1.68. This is a lower Mg/Al ratio than is typically found in hydrotalcite-group minerals, which are more commonly ∼2–3.^17^ This suggests either that an unusually Al-rich LDH phase has formed here, or that there is an additional Al-rich phase present intermixed with the bulk matrix. It may be possible that some of the AFm-structured material is finely dispersed through the bulk gel in addition to its presence in discrete crystallites as identified by SEM. However, a more likely explanation is the formation and intermixing of a disordered sodium aluminosilicate hydrate (N-A-S-H) type gel, as identified by Myers *et al.*^29^ in sodium silicate-activated slag cements, and this will be further addressed by NMR in the following section.

**Fig. 16:**
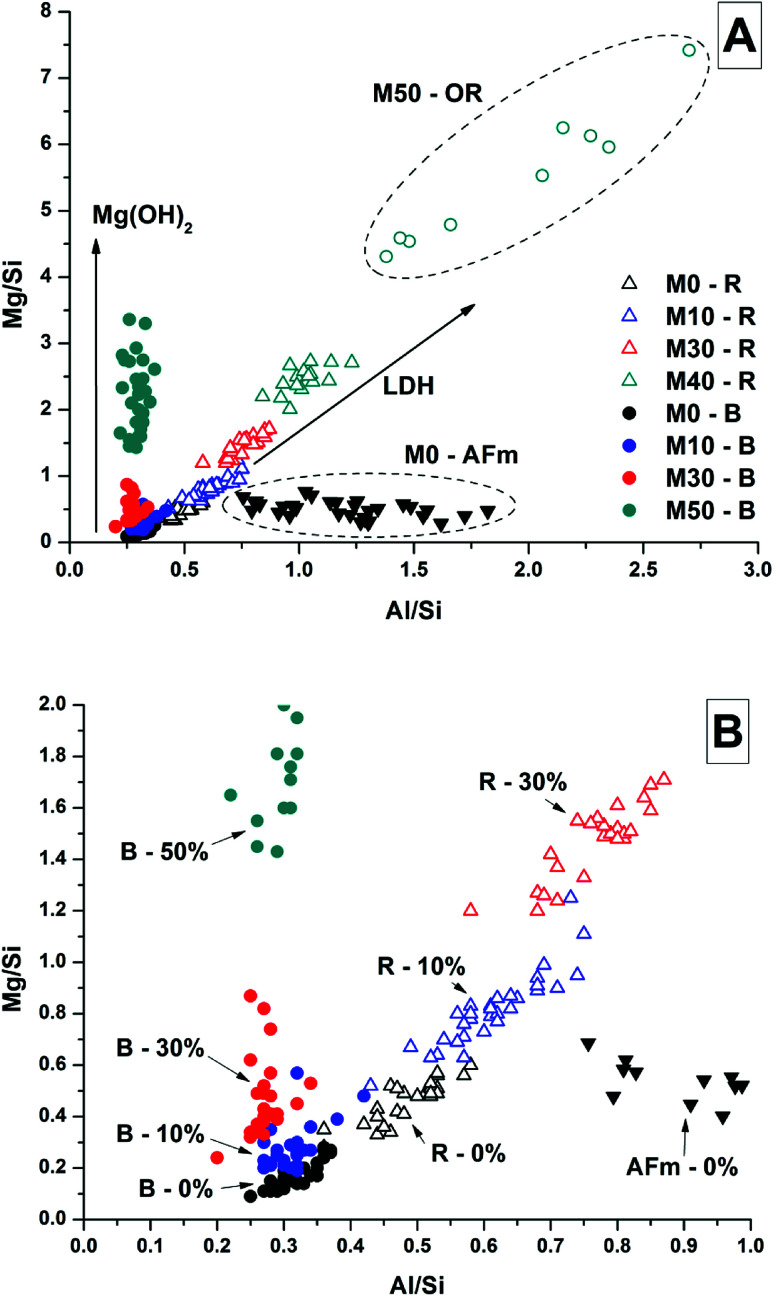
Plots of EDX spot map atomic ratios, comparing Mg/Si *vs.* Al/Si ratios of 18 months samples with up to 50% Mg(OH)_2_. (A) Full plot, (B) enlargement of the lower-left region. Abbreviations used: B = Bulk, R = Rim, OR = outer rim [r_o_], LDH = layered double hydroxide.

It was not possible to perform this type of quantitative analysis of Mg/Si *vs.* Al/Si ratios to determine phase compositions in samples with Mg(OH)_2_ additions, as the fine dispersion of Mg(OH)_2_ throughout the samples meant that not all of the Mg detected could be assigned to a hydrotalcite-like phase. Nonetheless, Fig. 16 shows the Mg/Si *vs.* Al/Si ratios for the bulk and rim EDX points in samples M0 – M50, along with the data points for the phase in M0 identified as the CO_2_-AFm as a comparison. Overall, the Mg content of both the bulk and the reaction rims in Mg(OH)_2_-containing samples appeared to increase, due to interference from dispersed Mg(OH)_2_ particles, which caused the bulk to appear rich in Mg, and also caused the reaction rim appear to have a higher Mg/Al ratio than its true value. Interestingly, the outer rim in the M50 sample showed a much higher Mg/Al ratio than the reacted slag rims in that sample. This mirrors the observations from the EDX maps, suggesting that this is almost completely a Mg-Al LDH phase, rather than a mix of C-(N-)A-S-H and MA-OH-LDH as was the case for the inner reaction rims, and for the slag rims observed in the other samples.

### 3.3. Solid state nuclear magnetic resonance spectroscopy

**Fig. 17:**
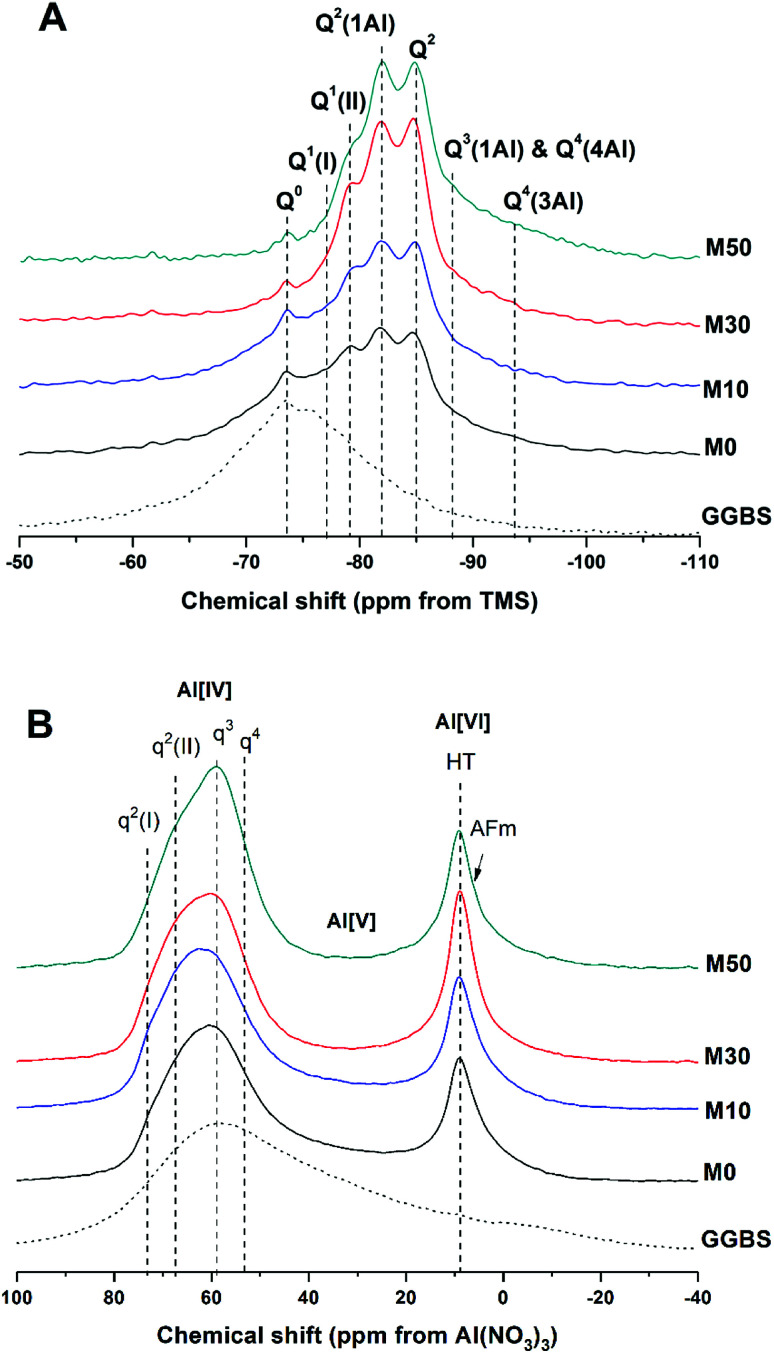
Solid state MAS NMR spectra of anhydrous GGBS and sodium carbonate activated slag binders after 18 months of curing, (A) ^29^Si, (B) ^27^Al.

**Fig. 18:**
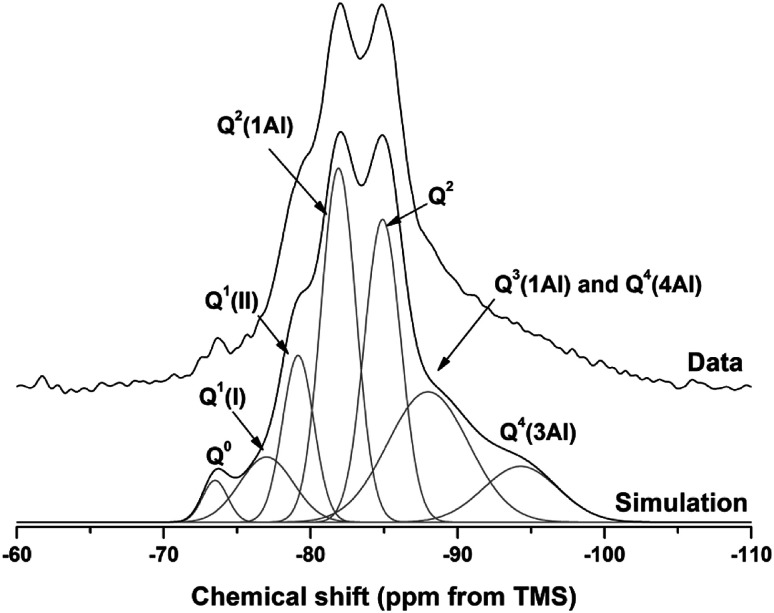
^29^Si MAS NMR spectrum for M50 after 18 months of curing. Simulation and constituent peaks are shown underneath the measured data. Peak assignments are detailed in Table 5.

The ^29^Si and ^27^Al MAS NMR spectra of 18 months-cured samples with different contents of Mg(OH)_2_ are displayed in Fig. 17, along with the spectrum of unreacted GGBS for reference. All spectra are normalised to integrated area for comparison. The incomplete, and potentially incongruent, dissolution of slag glass in sodium carbonate activated slag pastes hinders detailed deconvolution of the ^29^Si MAS NMR spectra. However, no residual slag was detected by SEM in the M50 sample (Fig. 12 and 13), and so this spectrum was deconvoluted (Fig. 18) and the resultant peak positions were used as the basis for interpretation of the other spectra.

The spectral deconvolution for sample M50 (Fig. 18) was conducted following, where possible, the peak assignments previously determined by Myers *et al.*^29^ for alkali activated slag binders, who interpreted this structure as a mixture of C-(N-)A-S-H and N-A-S-H type gels. The data presented show strong similarities to those for slag cements activated with both with sodium carbonate and sodium silicate activators.^6,22,69^ Four distinct peaks were readily identifiable within the ^29^Si MAS NMR spectra (Fig. 17A), along with a broad resonance centred at ∼−74 ppm associated with residual slag. Other peaks and broad shoulders were inferred from the deconvolution presented in Fig. 18. The Q^1^ region is split into two sites (−77.0 and −79.2 ppm), following the reasoning of Myers *et al.*^29^ who highlighted the potential existence of multiple Q^1^ environments due to differences in shielding by charge balancing Ca^2+^, H^+^ and Na^+^ cations. In general, as GGBS is replaced by Mg(OH)_2_, the broad resonance associated with the slag decreases in intensity. The proportion of Q^2^(1Al) to Q^2^ sites appears to remain constant, as both component peaks increase in intensity, consistent with the formation of longer chains in a cross-linked bulk C-(N-)A-S-H gel and more extensive dissolution of the slag at higher Mg(OH)_2_ replacement levels.

The deconvoluted ^29^Si MAS NMR spectrum for the paste with 50 wt% Mg(OH)_2_ (Fig. 18) had a lower proportion of Q^1^(II) and Q^2^(1Al), and a larger Q^2^ contribution, than is typically identified for alkali-activated slag systems.^22,28,69^ Although all the slag glass had reacted in sample M50, a minor Q^0^ peak at −73.5 ppm remained, which is tentatively attributed to residual ordered åkermanite-like regions^70^ from the slag which were less reactive than the glass (although not sufficiently crystalline to be identified by XRD), as also seen in all other ^29^Si MAS NMR spectra (Fig. 17A). The reduced intensity for the Q^2^(1Al) site indicates that the C-(N-)A-S-H gel contains a lower degree of Al substitution than silicate-activated slag binders,^22,29^ consistent with the Al being incorporated into hydrotalcite-like and C_4_AĈH_11_ phases previously identified *via* XRD (Fig. 4) and SEM analysis (Fig. 12).

The presence of a N-A-S-H-type gel, as postulated above, is supported by the existence of resonances upfield of that of the Q^2^ site. The resonance at −88.0 ppm has been assigned to both Q^3^(1Al) from cross linking within the C-(N-)A-S-H gel, and to a Q^4^(4Al) site from a N-A-S-H type gel. This cannot arise from a C-(N-)A-S-H gel alone, as the total resonance intensity at this frequency is much larger than is possible for a C-(N-)A-S-H gel (Table 5), which structurally can accommodate a maximum of 1 Q^3^(1Al) per 4 Q^2^(0,1Al) sites.^71^ The presence of a further resonance at −94.3 ppm, which is assigned to a Q^4^(3Al) site, provides additional evidence for the contribution from a N-A-S-H gel.

**Table 5:** Peak positions and widths (ppm) for deconvolution of ^29^Si MAS NMR data for M50 at 18 months [intensity as % area of integrated total fit]

	Q^0^	Q^1^(I)	Q^1^(II)	Q^2^(1Al)	Q^2^	Q^3^(1Al)/Q^4^(4Al)	Q^4^(3Al)
Intensity (%)	2	7	11	26	23	22	9
Centre	−73.5	−77.0	−79.2	−81.9	−84.9	−88.0	−94.3
FWHM	2.0	4.3	2.5	2.8	2.9	6.5	6.0

Although this sample (M50) contained a substantial proportion of Mg(OH)_2_, there was no evidence of the reaction of this Mg(OH)_2_ to form additional magnesium-containing phases according to FTIR, XRD or SEM as presented above. If an M-S-H type phase was formed, this would be expected to show distinct resonances between −86 and −89 ppm and between −93 and −98 ppm, depending on the M-S-H composition.^72,73^ Although these fall in the regions assigned to Q^3^(1Al)/Q^4^(4Al) and Q^4^(3Al) here, there was no evidence for the formation of these from any other techniques, and the fact that the atomic ratio correlation plots indicate that the additional disordered phase present is Al-rich rather than Mg-rich (*i.e.* N-A-S-H not M-S-H) supports this argument.

The resonances in the ^27^Al MAS NMR spectra (Fig. 17B) are split into Al[IV], Al[V] and Al[VI] regions. The Al[IV] region contains Al resonances from the unreacted glassy slag, along with the cross linking Al sites within the C-(N-)A-S-H gel, which combined form a broad feature centred around 60 ppm. Within the Al[IV] region, different q^*n*^ sites have been tentatively designated, denoting connectivity comparable to the Q^*n*^ notation for Si. Considering the results of Myers *et al.*^29^ for sodium silicate activated slag cements, two q^2^ sites are observed, corresponding to Al sites in a C-(N-)A-S-H gel. A resonance at 74 ppm has been reported as being due to the Al in bridging positions linked to Q^2^(1Al) silicon environments within a C-(N-)A-S-H gel, and would typically exhibit a sharp peak,^28^ but is not so prominent within these spectra. This is in agreement with the reduced prominence of the Q^2^(1Al) Si site in these samples (Fig. 17A) compared to the existing literature. The q^3^ site, which is the bridging crosslink site in C-(N-)A-S-H, is particularly prominent within these samples, and q^4^ sites are characteristic of N-A-S-H. This fits well with the identification of Q^4^(4Al) and Q^4^(3Al) sites within the ^29^Si MAS NMR spectra.

The Al[V] region shows only a minor Al environment in the range ∼45–20 ppm, present in the glassy slag fraction, which correspondingly disappeared in samples with higher contents of Mg(OH)_2_ replacement (M30 and M50) as the slag underwent reaction. The C-(N-)A-S-H here does now show a significant presence of Al(v) in its interlayer.

The Al[VI] region only displays a single broad resonance at 8–9 ppm, associated with a combination of MA-OH-LDH hydrotalcite-like structures and the hydrated calcium aluminate (postulated C_4_AĈH_11_) phase,^74,75^ which have similar chemical shifts and so overlap in the spectra. This made further elucidation of the signals in this region difficult, but the presence of both contributions is supported by the identification of both these phases in the XRD and SEM/EDX analysis.

The NMR analysis here demonstrates the presence of a highly cross-linked C-(N-)A-S-H gel, an N-A-S-H gel, and MA-OH-LDH and CO_2_-AFm phases. This is a complex system, with many phases which have the potential to immobilise or retain radionuclides if used in a waste immobilisation application. None of the key binding phases appear to have undergone any reaction or ionic substitution with Mg from the Mg(OH)_2_ which was added into these cements. There is ultimately no evidence presented here for the formation of a M-S-H or M-A-S-H gel, from either NMR, XRD or SEM, and this indicates encapsulation of Mg(OH)_2_ rather than its incorporation through chemical reaction.

## 4. Conclusions

This study has highlighted the hydration mechanisms and products of sodium carbonate activated slags, both with and without the addition of Mg(OH)_2_. A C-(N-)A-S-H/N-A-S-H assemblage, a hydrotalcite-like compound and a hydrous AFm-structured calcium carboaluminate phase were the major reaction products identified in all samples, with calcite increasingly formed as the slag was replaced with Mg(OH)_2_ and the Na_2_CO_3_/slag ratio thus increased. The hydrotalcite-like compound was identified as a carbonate-free layered double hydroxide, containing OH^−^ interlayer anions, in agreement with recent thermodynamic modelling for similar systems. Carbonate appears to be sequestered initially within gaylussite, which progressively dissolves and converts to calcite over time.

The added Mg(OH)_2_ appeared only to act as a filler and nucleation seed, reducing the time taken for major strength forming phases to develop, while undergoing little to no further reaction with any other components within the system. No magnesium bearing phases were identified as rims around the Mg(OH)_2_ particles, suggesting that the nucleation seeding primarily influenced C-(N-)A-S-H (and maybe N-A-S-H) gels.

Overall, a sodium carbonate activated slag matrix would be suitable for the encapsulation of Mg(OH)_2_-bearing wastes such as Magnox sludge. Little if any reaction would be expected to occur between the Mg(OH)_2_-bearing waste material and the cementitious matrix. The release of heat of reaction is accelerated when adding Mg(OH)_2_, though this does not increase the cumulative heat of reaction above that which is observed in Mg(OH)_2_-free samples. The slow hydration of these samples means they can be safely processed without risk of thermal runaway or heat-induced cracking in larger samples.

## Conflicts of interest

There are no conflicts of interest to declare.

## Supplementary Material
